# Tension Pneumocephalus After Right Dacryocystorhinostomy: A Rare but Potential Serious Complication

**DOI:** 10.7759/cureus.8635

**Published:** 2020-06-15

**Authors:** Sher Muhammad Sethi, Syed Ahsan Ali

**Affiliations:** 1 Internal Medicine, Aga Khan University Hospital, Karachi, PAK

**Keywords:** pneumocephalus, dacryocystorhinostomy

## Abstract

Tension pneumocephalus is a phenomenon in which air enters through dural injury in the brain and then expands and causes a mass effect. The injury can be due to any neurosurgical procedure, trauma, infection and/or neoplasm. A 63-year-old female known case of diabetes and hypertension had an elective procedure of right dacryocystorhinostomy present to the emergency department the very next day with a loss of consciousness. Urgent CT of the head showed air in the cranium (pneumocephalus). A radiological sign named “Mount Fuji” is classical for tension pneumocephalus. She was closely monitored and shows good clinical improvement allowing the neurosurgery team to avoid any intervention. The rationale to present this case is that to our knowledge, this is the first case in which tension pneumocephalus had occurred post-dacryocystorhinostomy. Due to the delicate region operated during eye surgeries, one should be more careful and vigilant.

## Introduction

Tension pneumocephalus is a phenomenon in which air enters through dural injury in the brain and then expands and causes a mass effect. The injury can be due to any neurosurgical procedure, trauma, infection and/or neoplasm [1-3]. It had diverse clinical manifestations and is mostly due to the mass effect produced by the air. Clinical features include headache, altered level of consciousness, seizures and focal neurological deficits [4].

To diagnose tension pneumocephalus, CT of the head is recommended which shows a classical radiological sign, named “Mount Fuji” sign [5]. Neurosurgical intervention is mostly required that includes needle aspiration, burr holes and craniotomy to relieve the mass effect [1]. There exists a benign variety of pneumocephalus which is a commonly encountered post-neurosurgical procedure. This benign pneumocephalus does not warrant urgent neurosurgical intervention and can be managed with close observation as air resorbs on its own [6].

The rationale to present this case is that to our knowledge, this is the first case in which tension pneumocephalus had occurred post-dacryocystorhinostomy. Ophthalmologists need to be more focused on operating this area as rare but a serious complication can exist.

## Case presentation

A 63-year-old female known case of diabetes and hypertension had an elective procedure of right dacryocystorhinostomy in December 2019 present to the emergency department the very next day with a loss of consciousness. It was sudden in onset, and there was no fever, headache, seizure, or any other associated symptoms. There were no emotional triggers, no physical exertion and no aural signs before the incident. The family did not notice any abnormal movements, tongue biting and incontinence. Past medical history was unremarkable for syncope, epilepsy, trauma or heart block. Home medications include insulin (for diabetes), angiotensin-converting-enzyme (ACE) inhibitors (for hypertension) and paracetamol for pain relief. Family history was insignificant for any syncope, epilepsy or arrhythmias. She is a housewife by profession, and there was no smoking or alcohol use in the past.

**Figure 1:**
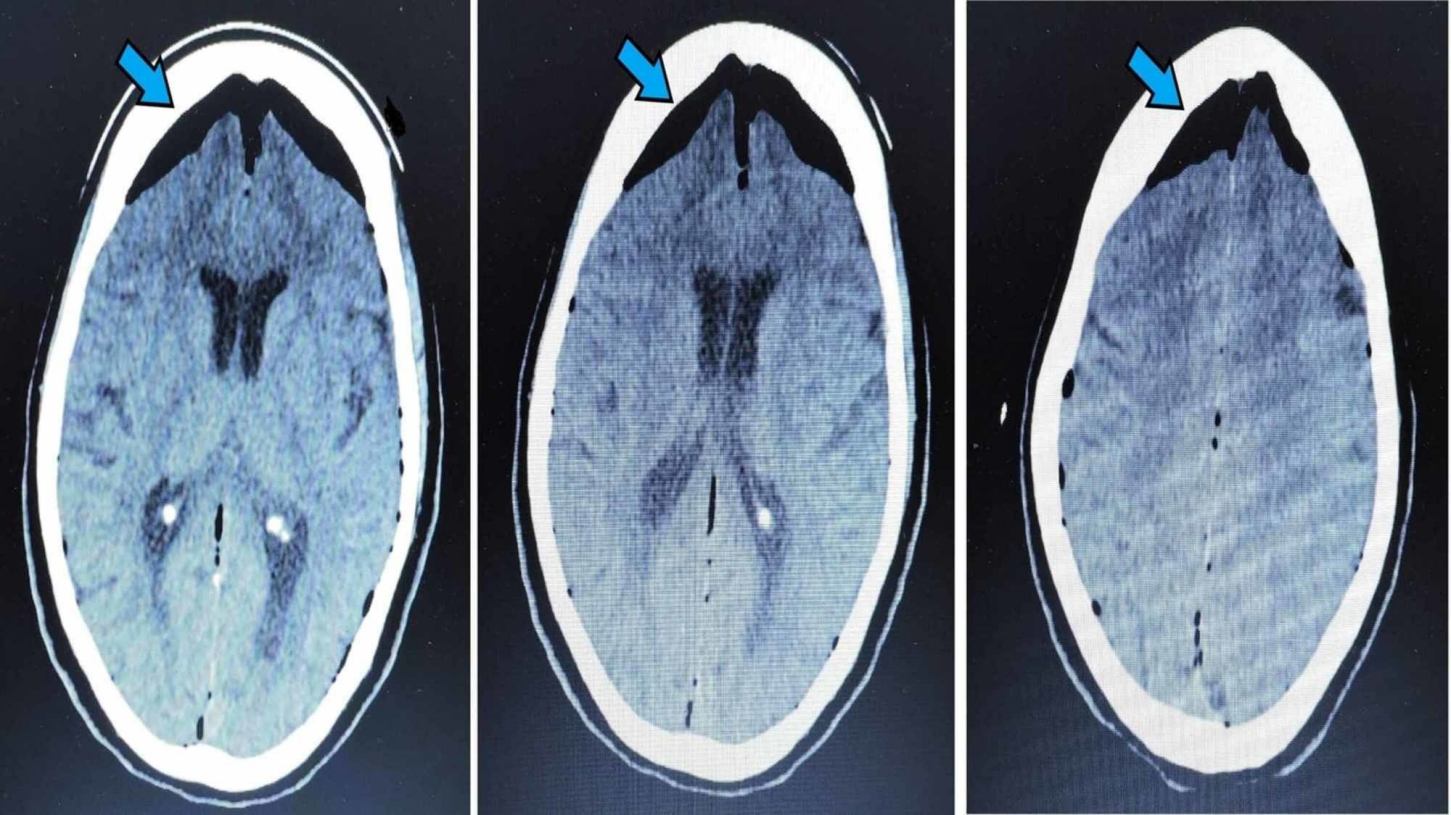
Mount Fuji Sign shown by the blue arrows

She was immediately rushed to the emergency department in an unconscious state and was intubated immediately. Her vitals showed blood pressure 125/65 mmHg, heart rate 105 beats per minute and blood sugar 265 mg/dL. The general appearance was unremarkable, chest auscultation showed normal vesicular breathing and there were no cardiac murmurs. The abdomen was soft, non-tender and no visceromegaly. Pupils were 4 mm in size and were reactive to light. Planters were downgoing bilaterally. Urgent CT of the head (Figure 1) showed air in the cranium (pneumocephalus). Arterial blood gas was done and showed pH 7.31, pCO_2_ 50 mmHg, pO_2_ 61 mmHg and HCO_3_ 24 mg/dl. Complete blood counts and electrolytes were normal. Urine was unremarkable and was negative for ketones. Echocardiography showed an ejection fraction of 55%. An electroencephalogram was done to rule out subclinical seizures.

**Figure 2:**
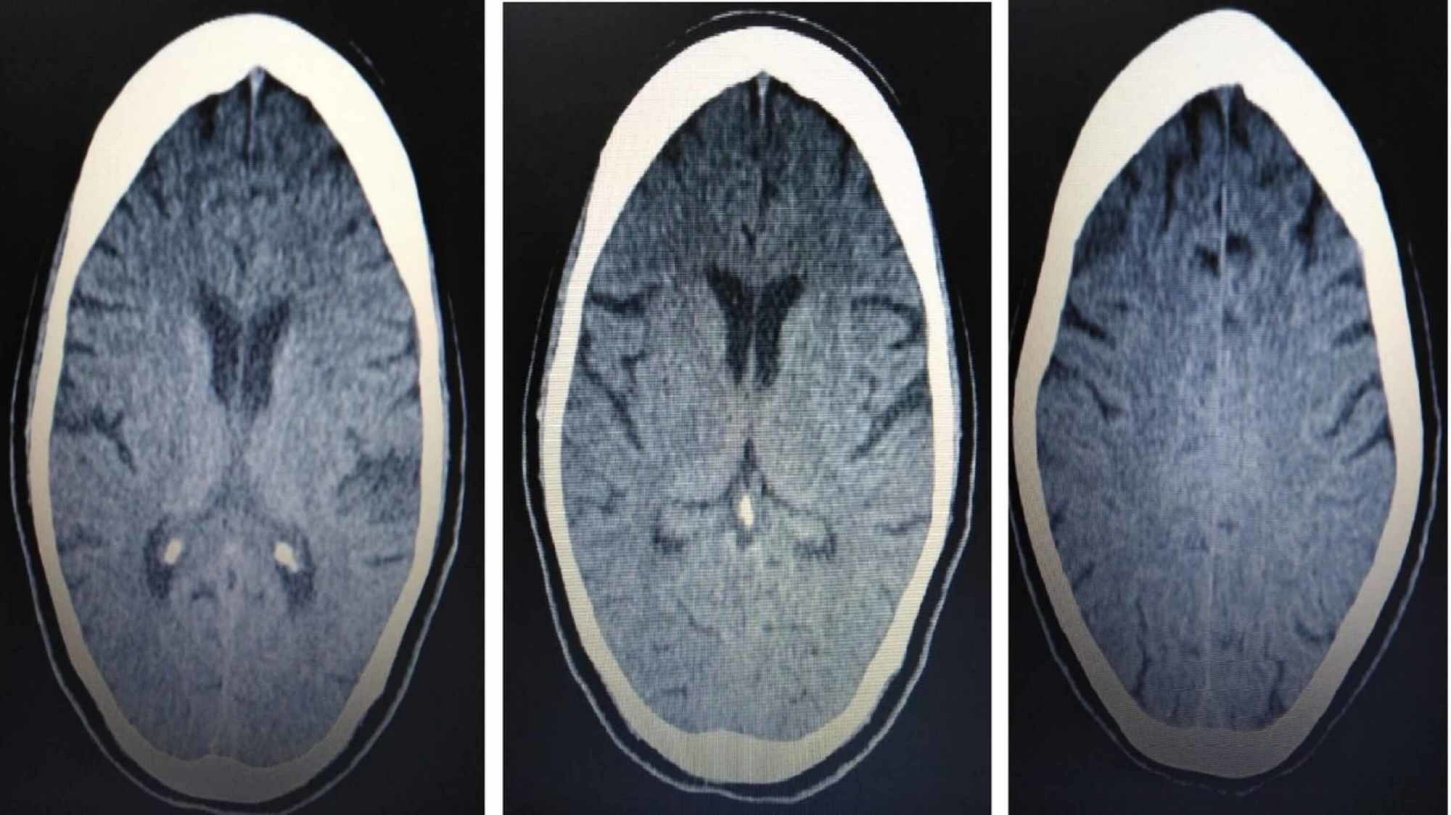
Resolution of air in cranium on follow-up scan

The neurosurgery and ophthalmology team was consulted and advised for keeping head end elevated at 45 degree and close monitoring. Surprisingly, she did not show any deterioration. It was decide to observe her. She improved clinically and was extubated. Post-extubation, she showed no focal deficit and had a normal gait. She was then discharged home. After one month, a follow-up scan was advised which shows complete reduction of air in the cranium (Figure 2). Therefore, the air resolved spontaneously and she did not require any intervention.

## Discussion

Tension pneumocephalus is the collection of air in the cranium that causes mass effect and produces neurological manifestations. Post-surgery especially neurosurgical procedures had been associated with tension pneumocephalus [3,7]. Neurosurgical interventions are one of the most common causes of this rare complication. In our patient, the right dacryocystorhinostomy was done one day before the onset of symptoms. There had been a case report of tension pneumocephalus following orbital exenteration [8]. Due to the proximity of skull bones during ocular procedures, there remains a possibility of injuring the skull bone and ending up in pneumocephalus. Though rare, this can be a potentially serious complication and warrant ophthalmologists to be more skilled and careful while performing these surgeries.

Due to increased intracranial pressure caused by air, the neurological consequences can be devastating. The features range from a simple headache to an acute loss of consciousness and neurological deficits [9]. Fortunately, in our case, the patient only had a sudden loss of consciousness and no focal deficits or seizures were noticed. All other probable causes of unconsciousness were excluded. CT of the head demonstrates the Mount Fuji sign. The sign shows subdural free air in the cranium compressing the frontal lobes and widening the interhemispheric fissure between the two lobes. It resembles the silhouette of Mount Fuji, hence named [5].

Management of tension pneumocephalus is mostly neurosurgical intervention. Various methods are used to resolve the air by needle aspiration, burr holes, craniotomy and closure of dural defects. Besides this, post-surgical pneumocephalus is occasionally associated with benign nature [1]. Correlation of imaging features to identify intracranial pressure is of immense importance in guiding towards management. Surgical intervention versus close observation can be decided on that basis. Avoidance of hyperventilation and use of normobaric hyperoxia are the medical methods used to treat and cause gradual resorption of air with time [10]. Similarly in our case, the CT scan did not show significant mass effect and only close monitoring was done. High airway pressure during ventilation was avoided, and the patient was gradually weaned off and kept on high flow oxygen to encourage air resolution. Within the next few days, we noticed a great recovery of the patient and she was discharged home comfortably.

This was a unique case for our medical team. An informed consent and ethical exemption was applied from the institution. We had a thorough literature search and was unable to find any correlation between tension pneumocephalus and dacryocystorhinostomy. Therefore, it was important to emphasize this case report to educate other health care workers. Due to the delicate region operated during eye surgeries, one should be more careful and vigilant.

## Conclusions

Tension pneumocephalus is rare but can be a grim complication of neuro-ocular surgeries. Surgeons should be vigilant in operating these delicate areas, and physicians should be cautious in early diagnosing and prompt management to avoid any further complications. Take home message is for young doctors and residents to identify the Mount Fuji sign on CT scan immediately.
